# Vasculitic Moyamoya syndrome in a patient with paroxysmal nocturnal hemoglobinuria: A case report and literature review

**DOI:** 10.1111/cns.13891

**Published:** 2022-07-11

**Authors:** Fangfang Li, Mingrong Xia, Hongyu Li, Dan Li, Dan Guo, Weizhou Zang, Jiewen Zhang

**Affiliations:** ^1^ Department of Neurology, Henan Provincial People's Hospital Zhengzhou University People's Hospital Zhengzhou China

Dear Editor,

Moyamoya syndrome **(**MMS) is a chronic and progressive occlusive cerebrovascular disease characterized by steno‐occlusive changes at or around terminal carotid arteries or the initial segments of the anterior/middle cerebral arteries accompanied by the development of fragile collateral network, which is often secondary to an underlying acquired or inherited condition. Paroxysmal nocturnal hemoglobinuria (PNH) is an acquired hematopoietic stem cell genetic mutation disease manifesting as hemolytic anemia, marrow failure, smooth muscle dystonia, and thrombosis.^1^ MMS secondary to PNH is rarely reported in the literature.^1^, ^2^, ^3^, ^4^ However, to the best to our knowledge, there has been no direct evidence about the vasculitic moyamoya changes of intracranial arteries in the situation of PNH. Here, we provide the direct evidence using intracranial high‐resolution vessel wall imaging (vw‐MRI) to present this phenomenon in our patient and discuss its possible cause and treatment.

A 42‐year‐old woman was admitted to Henan Provincial People's Hospital with a 20‐day history of clumsy movement of her right hand and slurred speech for 1 day. Initially diffusion weighted imaging (DWI) of the brain showed acute ischemic stroke in left frontoparietal lobe when she showed clumsy movement of her right hand 20 days earlier (Figure 1A). In the meanwhile, magnetic resonance angiography (MRA) indicated stenosis in the proximal part of bilateral middle cerebral artery (MCA), moderate‐to‐severe stenosis in left internal carotid artery (ICA) and A1 segment of left anterior cerebral artery (ACA) (Figure 1B). Laboratory examination indicated moderate macrocytic anemia (Hemoglobin 6.6 g/dL, mean corpuscular volume 108 fL) and high values of lactate dehydrogenase (LDH, 1672 U/L). The patient denied any family history of cerebrovascular disease and no obvious vascular risk factors were found. However, a history of PNH (flow cytometry showed CD55 negative equal to 21.41% of the erythrocytes, CD59 negative equal to 28.16% of the erythrocytes, CD55 negative equal to 86.42% of the granulocytes, CD59 negative equal to 84.73% of the granulocytes) was reported and hemolytic attacks occurred occasionally. Aspirin (100 mg per day) and rosuvastatin (10 mg per day) was administered and her symptoms improved gradually. One day before her admission, the patient presented with weakness of her right upper limb and slurred speech. In addition, the second brain DWI revealed multiple acute cerebral infarctions in right frontal regions and extended ischemic lesions in left frontoparietal lobe (Figure 1C). On physical examination, power was 2/5 in right upper limb and 4/5 in the other three limbs. During the second day, laboratory examinations demonstrated the patient had slight macrocytic anemia with a hemoglobin of 8.5 g/dL with the mean corpuscular volume of 116.4 fL, LDH 1702 U/L, total bilirubin 31.8umol/L, direct bilirubin 10.5 μmol/L and indirect bilirubin 21.3 μmol/L. Dual antiplatelet therapy (Aspirin 100 mg per day and clopidogrel 75 mg per day) and intensive lipid‐lowering treatment (rosuvastatin 20 mg per day) were started. Vw‐MRI was then conducted and revealed smooth, homogeneous, concentric arterial wall thickening and enhancement in left ICA. Meanwhile, vw‐MRI also showed steno‐occlusive lesions in the petrous segment, distal communicating segment of left ICA and formation of moyamoya vessels (Figure 2A–D). The patient's situation worsened gradually during hospitalization. In the eleventh day on her admission, deteriorative mental status leading to somnolence, aphasia and recurrent intermittent episodes of dystonia were observed. Physical examination showed active tendon reflexes in all extremities and bilateral Babinski signs were positive. Routine blood test was performed immediately and showed that hemoglobin dropped to 6.7 g/L. Brain magnetic resonance imaging (MRI) was repeated on twelfth day after admission and manifested bilateral frontotemporal lobes and basal ganglia acute ischemic lesions (Figure 2E–F). Considering the exacerbation of patient's cerebral infarction, intermittent episodes of hemolytic events, and vasculitic changes of intracranial arteries, intravenous methylprednisolone (500 mg/d for 3 days) was prescribed, with doses decreased gradually over time. Following the treatment, her consciousness started to improve and could make eye contact with her families. Intermittent episodes of dystonia tapered as well. Twenty‐fourth day after her admission, the patient developed difficulties in breathing and decreased blood oxygen saturation. Pulmonary artery computed tomography angiography (CTA) was then conducted and displayed multiple thrombosis in bilateral pulmonary arteries (Figure 2I). In the meanwhile, ultrasound found thrombosis in the intermuscular vein of the right lower extremity. Then antiplatelet therapy was replaced by low molecular weight heparin (5000 IU, Q12H, IH) and the patient's respiratory symptoms improved soon. Thirty‐second day on her admission, brain MRI was reexamined, and no new lesions were found (Figure 2G–H). Pulmonary arteries thrombus also disappeared on forty‐third day after admission (Figure 2J). The patient could partially cooperate with the instructions and hemoglobin raised to 8.9 g/L when discharged from hospital.

**FIGURE 1:**
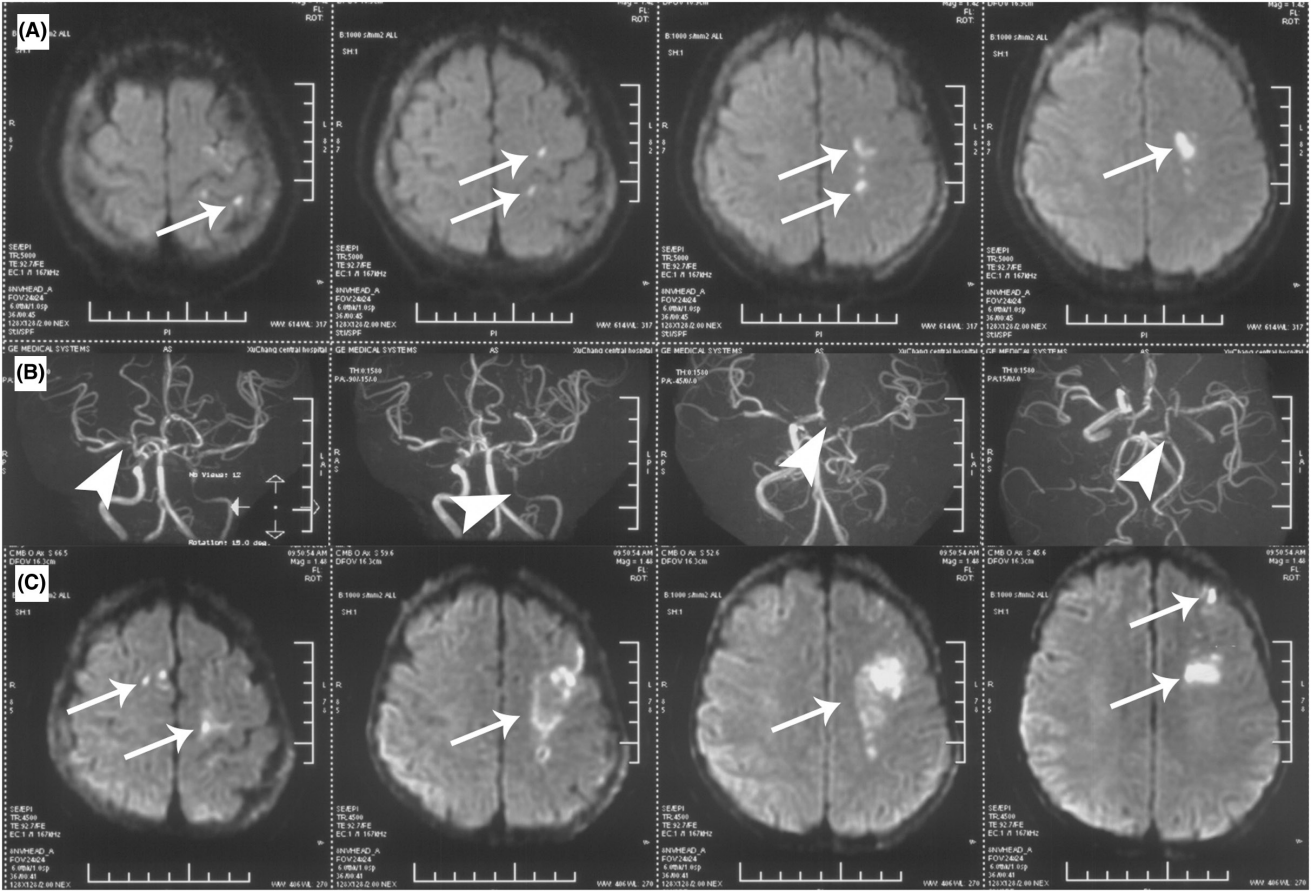
(A) Initially axial DWI of brain showed acute infarction in left frontoparietal lobe (long white arrow). (B) MRA demonstrated vascular stenosis in the proximal part of bilateral MCA, moderate to severe stenosis in left ICA, A1 segment of left ACA and proximal segment of posterior cerebral artery (PCA) (short white arrow). (C) The second axial brain DWI revealed multiple acute cerebral infarctions in right frontal regions and extended ischemic lesions in left frontoparietal lobe (long white arrow)

**FIGURE 2:**
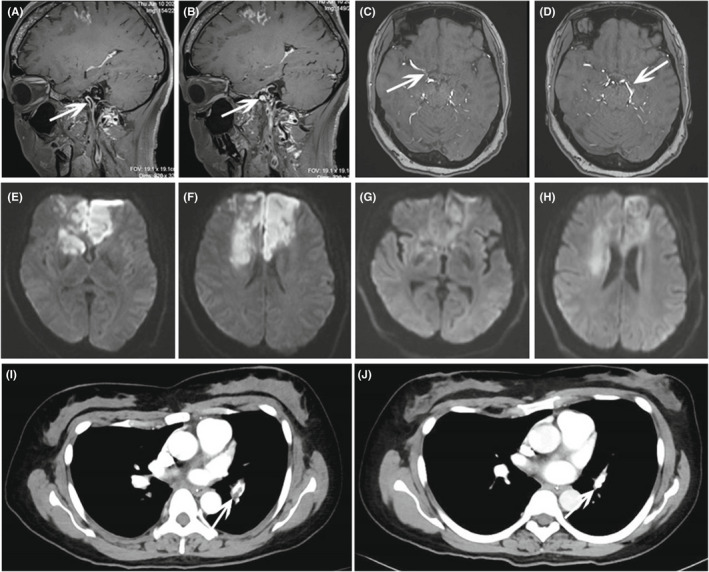
(A, B) Sagittal vw‐MRI showed smooth, homogeneous, concentric arterial wall thickening and enhancement in left ICA. (C, D) Axial MRA revealed the development of fragile collateral network. (E, F) Bilateral frontotemporal lobes and basal ganglia acute ischemic lesions were presented on twelfth day after admission. (G, H) Brain MRI was performed on thirty‐second day after admission and no new lesions appeared. (I) Representative image of pulmonary CTA showed thrombosis in left pulmonary arteries. (J) Pulmonary CTA was repeated before discharge and thrombosis in left pulmonary arteries was vanished

However, we knew from a telephone follow‐up that she died suddenly at home 2 months later.

## DISCUSSION

We provide a vw‐MRI image that directly supports vasculitic moyamoya changes of intracranial arteries in the situation of PNH for the first time, as far as we know. In our case report, the patient was diagnosed with PNH 3 years before her cerebral artery thrombosis, and hemolytic attacks occurred from time to time. Except PNH, the young patient has no family history of cardiovascular diseases or cerebrovascular risk factors. The first cerebral ischemic attack and subsequent stroke progression were both accompanied with hemolytic events (a reduction of hemoglobin as well as an increase of LDH and indirect bilirubin), which suggest a possible causal relationship between PNH and cerebral infarction. Next, vw‐MRI showed homogeneous, concentric arterial wall thickening and enhancement in terminal portions of left ICA and proximal segment of MCA, which indicated vasculitic moyamoya vessels in the patient.^5^ As complement activation, increased free hemoglobin, endothelial dysfunction and inflammatory reaction are the main pathological mechanisms of PNH, while the abovementioned pathological mechanisms also play major roles in vascular inflammation and heme can cause vascular inflammation followed by vascular obstruction,^6^ we presume that the patient's vasculitic moyamoya syndrome may be a result of PNH.

Paroxysmal nocturnal hemoglobinuria is a rare acquired bone‐marrow disease featured by deficiency of glycosylphosphatidylinositol (GPI)‐anchored complement regulatory proteins such as CD55 and CD59 on membrane of erythrocytes, leading to an increased sensitivity of red blood cells to membrane attack complex. It characteristically presents as hemolytic anemia, hemoglobinuria and intravascular thrombosis. Arterial thrombosis is an infrequent complication of PNH, accounting for approximately 15% of total thromboembolism events, which located primarily in cerebral vasculature (90.7%).^7^ To date, there were only four reports which described moyamoya phenomenon in PNH patients.^1^, ^2^, ^3^, ^4^ Among these patients, their ages ranged from 9 to 52 years old, and there were two males and two females. Except one little boy who received eculizumab treatment and had a good prognosis, the remaining three patients all presented with recurrent cerebral infarction and responded differently to antiplatelet therapy, which were detailed depicted in Table 1. However, none of these reports provided direct evidence about the vasculitic moyamoya changes of intracranial arteries in PNH patients. In our case report, except the vasculitic moyamoya changes, the patient also presented with multiple cerebral infarction, pulmonary artery thrombosis, as well as intermuscular vein of the right lower extremity. Antiplatelet therapy seems cannot stop the progression of thrombosis further certified hypercoagulable state in PNH,^6^ which reminds clinical physicians that anticoagulant remedy may be a superior choice in PNH patients with thrombosis event (if no contraindication). Besides, as cerebral blood flow is an important marker for clinical prognosis of moyamoya syndrome and sensitive to small blood pressure changes,^8^ it is necessary to monitor cerebral blood flow and maintain a strict blood pressure control in daily medical work.

**TABLE 1:** Clinical characteristics of patients with moyamoya syndrome and PNH reported in the literature

Patient	Age	Gender	Duration of disease	Hemoglobin concentration when neurological symptoms occurred (g/L)	Treatment	Follow‐up
Mugikura et al^3^	52	Female	5 months	88, 77	Antiplatelet	2 years. No additional infarction, and no progression of the steno‐occlusive lesions
Lin et al^2^	11	Female	1 year and 10 month	40, −, −	Prednisolone, coumadin, and washed red blood cell transfusion	No follow‐up information
Yen et al^4^	9	Male	‐	76	Eculizumab	No cerebrovascular accidents recurred
Cheng et al^5^	21	Male	12 years	50, 73	Aspirin, prednisolone, and washed red blood cell	1 year. Lost the ability to work for deteriorating weakness of the right limbs.
Our patient	42	Female	3 years	66, 85, 67	Dual antiplatelet therapy, prednisolone, washed red blood cell. Then antiplatelet therapy was replaced by anticoagulant therapy soon	2 months. Died at home

For the treatment of PNH associated vasculitic moyamoya syndrome, complement inhibitor such as Eculizumab to cope with PNH and reduce PNH relevant complications is the first choice,^9^ corticosteroid or immunosuppressive therapy may be an alternative strategy.^10^ Surgical revascularization seems to be a more advanced and effective option for ischemic moyamoya syndrome.

## CONFLICT OF INTEREST

The authors declare no conflict of interest.

## CONSENT FOR PUBLICATION

Written informed consent was obtained from the patient for publication of this case report and any accompanying images. A copy of the written consent is available for review by the editor of this journal. All authors agreed to publication.

## Data Availability

Data sharing is not applicable to this article as no datasets were generated or analyzed during the current study.
